# Erythropoietic response to oral iron in patients with nondialysis-dependent chronic kidney disease in the FIND-CKD trial

**DOI:** 10.5414/CN109198

**Published:** 2017-10-26

**Authors:** Iain C. Macdougall, Andreas H. Bock, Fernando Carrera, Kai-Uwe Eckardt, Carlo Gaillard, David Van Wyck, Yvonne Meier, Sylvain Larroque, Amandine Perrin, Simon D. Roger

**Affiliations:** 1 Department of Renal Medicine, King’s College Hospital, Denmark Hill, London, UK,; 2 Department of Nephrology, Kantonsspital Aarau, Aarau, Switzerland,; 3 Eurodial, DaVita, Leiria, Portugal,; 4 Department of Nephrology and Hypertension, University of Erlangen-Nürnberg, Erlangen, Germany,; 5 Department of Nephrology, University Medical Center Groningen, University of Groningen, Groningen, The Netherlands,; 6 DaVita Inc., Denver, CO, USA,; 7 Vifor Pharma, Glattbrugg, Switzerland,; 8 Renal Research, Gosford, NSW, Australia;; *Members of the Ferinject® assessment in patients with Iron deficiency anemia and Non-Dialysis dependent Chronic Kidney Disease (FIND-CKD) study group are listed in the Appendix.

**Keywords:** ferritin, hemoglobin, oral, iron, nondialysis, supplement

## Abstract

Aims: To evaluate erythropoietic response rates to oral iron over time in iron-deficient anemic patients with nondialysis-dependent chronic kidney disease (ND-CKD). Materials and methods: FIND-CKD was a 1-year, randomized, multicenter trial of iron therapy in patients with ND-CKD, anemia, and iron deficiency, without erythropoiesis-stimulating agent (ESA) therapy. Patients with active infection or C-reactive protein > 20 mg/L were excluded. In this post-hoc analysis, response was defined as ≥ 1 g/dL increase in hemoglobin (Hb) from baseline, before initiation of alternative anemia therapy (i.e., ESA, transfusion, or intravenous iron). Results: 308 patients received oral iron (200 mg elemental iron/day). Mean (SD) Hb at baseline was 10.4 (0.7) g/dL. At week 4, Hb data were available from 292 patients without alternative anemia therapy: 63/292 (21.6%) showed a response. Among the 229 nonresponders at week 4, 48.8% showed a cumulative response on ≥ 1 occasion by week 52 (11.1%, 19.9%, 25.9%, and 28.7% had a response at weeks 8, 12, 24, and 52, respectively), and 27.9% had received alternative iron therapy by week 52. Baseline levels of Hb, ferritin, and transferrin saturation were lower in responders than in nonresponders. Neither concomitant medication nor adherence (as assessed by medication count) was substantially different between early responders and nonresponders. Conclusion: Four weeks after starting oral iron therapy, only 21.6% of anemic patients with ND-CKD and iron deficiency showed an Hb increase of at least 1 g/dL. Among early nonresponders, < 30% responded at any subsequent time point. Earlier consideration of alternative therapy could improve anemia management in this population.

## Introduction 

Iron deficiency is an important contributory factor in the pathogenesis of anemia in patients with nondialysis-dependent chronic kidney disease (ND-CKD). When thresholds for iron parameters are applied that have been established in dialysis patients, iron deficiency affects ~ 60% of male and 70% of female ND-CKD patients [1]. There are several causes of inadequate iron availability in ND-CKD, including poor appetite and low dietary intake of iron, restricted gastrointestinal absorption of iron due to increased hepcidin levels, and an increased tendency of bleeding in the gastrointestinal tract [2, 3]. Identification of iron deficiency should prompt an assessment of dietary adequacy, overt or occult causes of blood loss, and medications that can interfere with iron uptake, and iron supplementation is required in most cases. The Kidney Disease Improving Global Outcomes (KDIGO) recommendations suggest that iron therapy should be used in patients with ND-CKD to correct iron deficiency, either alone or prior to starting erythropoiesis-stimulating agent (ESA) therapy [4]. 

Oral iron therapy is often used in this setting since it is inexpensive and convenient [5, 6 ]. However, gastrointestinal side-effects are frequent, and rapid iron repletion is by no means assured since the bioavailability of iron from oral preparations is low and varies widely. Numerous factors contribute to this variability. Elevated hepcidin levels induced by the chronic inflammatory state of uremia [7] inhibit intestinal absorption of iron [8], but the extent of hepcidin upregulation differs substantially between individuals [9]. Absorption from oral iron preparations is also influenced by concomitant food or by co-medications such as calcium carbonate, phosphate binders, or proton pump inhibitors. Genetic variants on iron-related genes also appear to influence the bioavailability of iron from oral preparations [10]. The response to oral iron is relatively slow compared to intravenous (IV) iron supplementation [11, 12], with an erythropoietic response to oral iron monotherapy in anemic patients with ND-CKD (defined as an increase in hemoglobin (Hb) of at least 1 g/dL) reported in as few as 14% of cases by 5 weeks [13] and ≤ 30% by 7 weeks [14, 15]. In patients who are unable to absorb adequate oral iron, or if more rapid anemia correction is required, IV iron can be initiated. Therefore, it would be helpful to understand what proportion of early nonresponders may respond later and, ideally, to predict which patients are unlikely to ever respond. Such information could help to reduce unnecessary delay in switching therapy and correcting anemia. 

FIND-CKD was a large, multicenter 1-year trial of iron therapy in patients with ND-CKD who were not receiving ESA therapy [16]. Patients received either IV ferric carboxymaltose (FCM) with two different ferritin target levels or a regimen of twice-daily oral ferrous sulfate. A post-hoc analysis of patients randomized to the control arm was undertaken to establish response rates to oral iron therapy over time. Data from the intervention groups, in which patients received FCM, are shown for comparative purposes in a descriptive manner. 

## Materials and methods 

FIND-CKD was a 56-week, open-label, multicenter, prospective, randomized, three-arm study conducted across 193 sites in 20 countries (ClinicalTrials.gov NCT00994318). The study methodology has been published previously [17]. 

The study population comprised adult patients with ND-CKD. Key eligibility criteria were ≥ 1 Hb level between 9 and 11 g/dL, with any ferritin level < 100 µg/L (or < 200 µg/L with transferrin saturation (TSAT) < 20%), within 4 weeks of randomization, and estimated glomerular filtration rate (eGFR) ≤ 60 mL/min/1.73m^2^. No ESA was to have been given in the 4 months prior to randomization. Patients with a documented history of discontinuing oral iron therapy due to significant gastrointestinal distress, or who had known active infection, C-reactive protein (CRP) > 20 mg/L or overt bleeding were excluded, as were patients with active malignancy. 

Patients were randomized in a 1:1:2 ratio to one of three groups: IV FCM targeting a higher ferritin level (400 – 600 µg/L) or a lower ferritin level (100 – 200 µg/L), or oral iron. Oral iron therapy consisted of commercially-available ferrous sulfate at a dose of 304 mg (200 mg elemental iron) daily to week 52 (Plastufer^®^ 100-mg elemental iron capsules (Haupt Pharma Münster GmbH, Münster, Germany, and Valeant Pharmaceuticals GmbH, Munich, Germany). During the first 8 weeks post randomization, patients were not to receive alternative anemia management (ESAs, blood transfusion, or any anemia therapy other than study drug) unless there was an absolute requirement. Subsequently, ESAs and other therapies were permitted if Hb was < 10 g/dL. Degree of adherence to oral iron therapy was assessed for the overall study period by counting returned unused capsules at each visit ((number of tablets dispensed – number of tablets returned)/(2 × (date returned – date dispensed)) × 100). 

Patients were categorized as “responders” or “nonresponders” based on data collected at the week-4 visit. Response was defined as an increase in Hb of ≥ 1 g/dL vs. baseline. Hb data were censored after an alternative anemia therapy (e.g., ESA, blood transfusion, or alternative iron product) was started or after the study or study drug was discontinued. Data were analyzed for all patients with Hb values at baseline and at the relevant post-baseline visit. Centrally-assessed values were used where available, with local readings used if central data were unavailable at any particular time point. This substantially expanded the pool of patients for analysis compared to restricting it only to patients with central values, a difference largely accounted for by missing central values at baseline. Baseline characteristics were compared between (a) the population in whom locally-read values were used in the event of missing centrally-read values and (b) only those patients in whom centrally-read Hb values were available. The two approaches were similar, based on calculation of 95% confidence interval (CI) values for demographic and baseline characteristics (Supplemental Table 1), reinforcing the validity of including locally-read values where necessary. 

Analyses are based on the intent-to-treat (ITT) population, consisting of patients who received at least one dose of randomized treatment and who attended at least one post-baseline visit. The Kaplan-Meier method was used to estimate time to event (i.e., Hb response) data, with groups compared using the log-rank test. 

## Results 

**Supplemental Figure 1.**
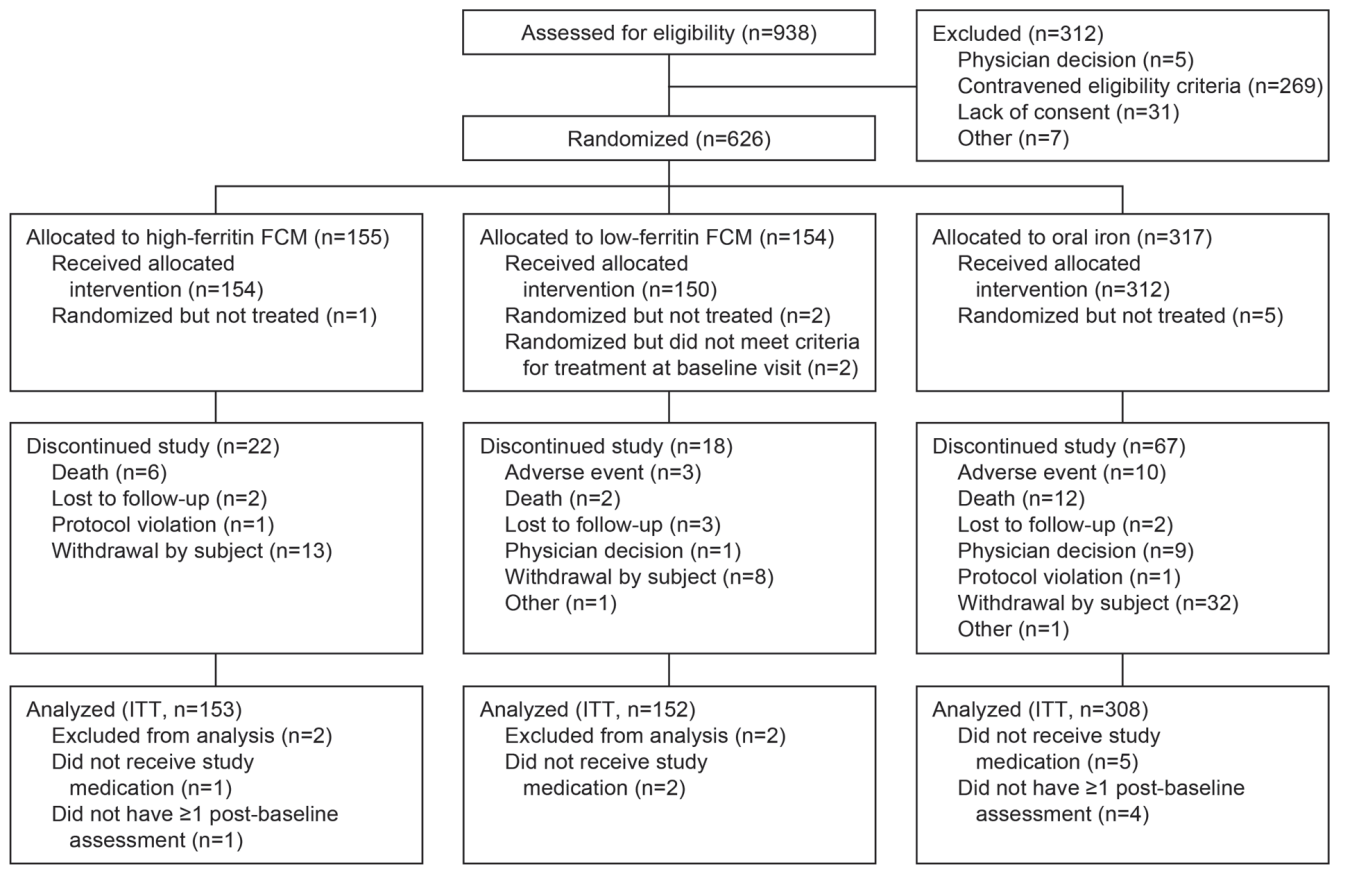
Patient disposition.

In total, 626 patients were randomized (317 oral iron, 155 high-ferritin FCM, 154 low-ferritin FCM), of which 613 met the criteria for inclusion in the ITT population (308 oral iron, 153 high-ferritin FCM, 152 low-ferritin FCM) (Supplemental Figure 1). Of the 308 patients in the oral-iron arm of the ITT population, the study was completed by 250 patients, with the most frequent reasons for discontinuation being withdrawal by the subject (n = 25), death (n = 12), adverse events (n = 9), and decision by the physician (n = 8) (Supplemental Figure 1). 

The mean (SD) age in the oral iron cohort was 69.3 (13.4) years, and the majority of patients (62.3%) were female. Mean (SD) Hb was 10.4 (0.7) g/dL (based on centrally-recorded values), with a mean ferritin value 57.3 (42.4) μg/L and a mean TSAT value 15.5 (7.6)%. Hepcidin data were available in a subset of 35 patients (mean (SD) 2.30 (2.01) nmol/L). Use of medications that potentially interfere with uptake of iron included antacids (12.7%, n = 39), H_2_ blockers or proton pump inhibitors (50.0%, n = 154), and the phosphate binders sevelamer or lanthanum carbonate (2.6%, n = 8). During treatment, mean (SD) adherence to oral iron therapy was 88.2 (18.4)%, with 230 patients (74.7%) showing adherence rates in the range of 80 – 100%. A total of 81/308 patients (26.3%) started alternative anemia therapy. 

**Figure 1.**
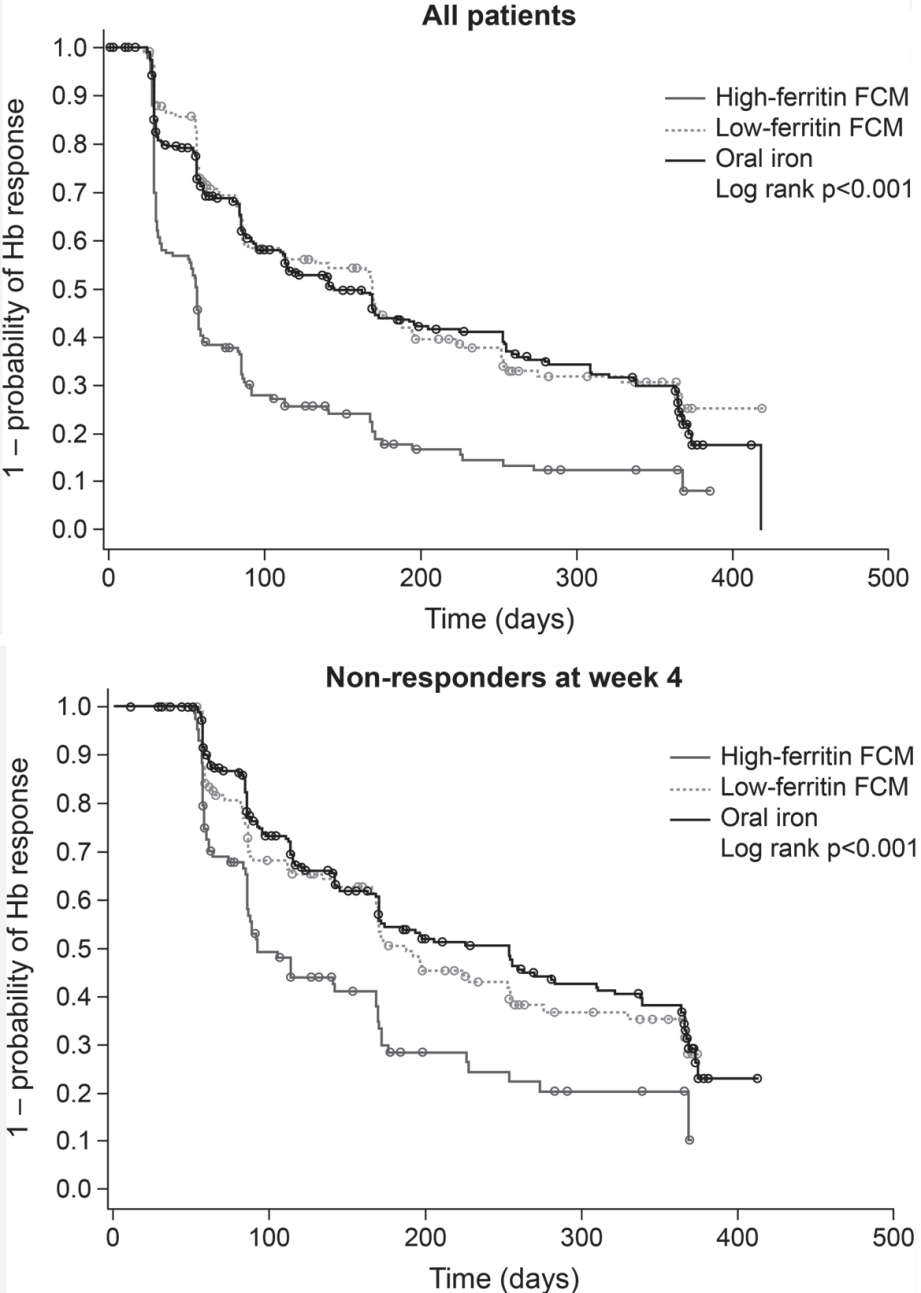
Kaplan-Meier plots of time to first Hb response in (a) all patients (b) patients who were not responders at week 4. FCM = ferric carboxymaltose.

Among the 308 patients analyzed in the oral-iron group, Hb levels were available at baseline and week 4 in 292 patients (either centrally- or locally-recorded values). The rate of Hb response by week 4 – defined as an Hb increase of ≥ 1 g/dL from baseline to the week-4 visit and excluding patients who started alternative anemia management – was 21.6% (63/292). The other 229 patients were “nonresponders”. For comparison, the response rates at week 4 in the high-ferritin and low-ferritin FCM groups were 40.9% (61/149) and 13.9% (20/144), respectively. Kaplan-Meier analysis showed a significant difference between the three groups in time to first response from baseline (Figure 1a). The median time to first response was 57 days (95% CI 34, 58) in the high-ferritin FCM group, 169 days (89, 195) in the low-ferritin FCM group, and 145 days (113, 185) in the oral-iron group. 

**Figure 2.**
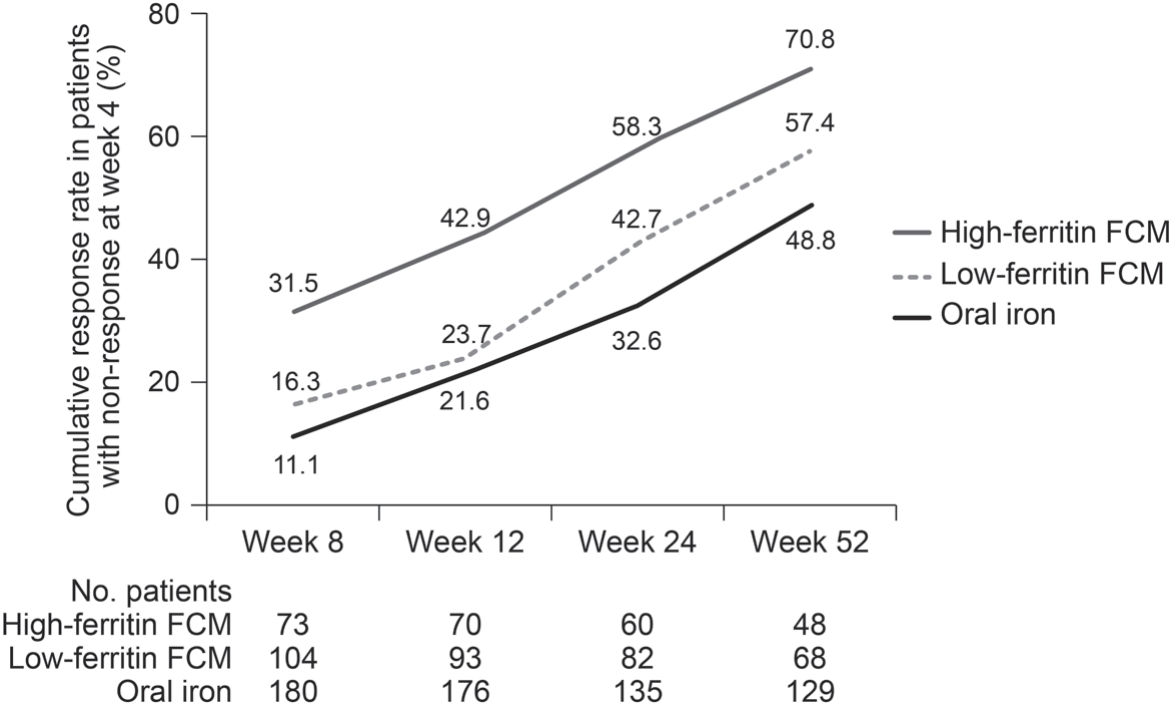
Cumulative response to iron therapy by weeks 8, 12, 24, and 52 in patients who were nonresponders at week 4. FCM = ferric carboxymaltose.

**Figure 3.**
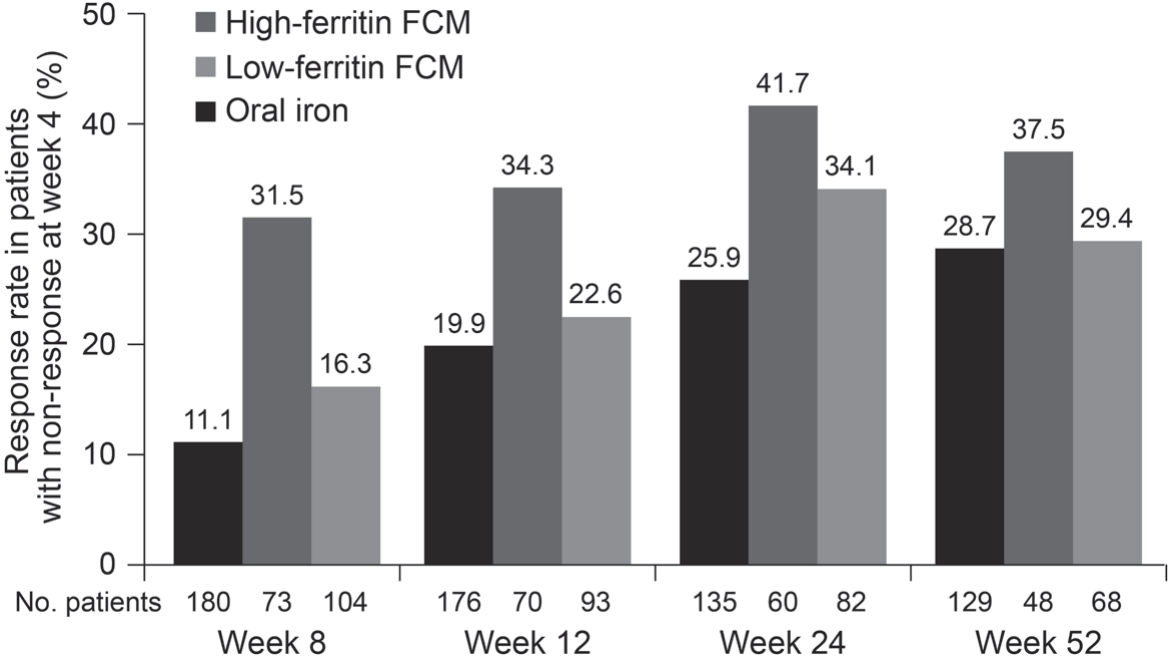
Response to iron therapy at weeks 8, 12, 24, and 52, in patients who were nonresponders at week 4. FCM = ferric carboxymaltose.

Among patients who did not respond to oral iron at week 4, the cumulative rate of response by week 52 (i.e., the proportion of patients who first showed an increase in Hb of ≥ 1 g/dL at any point after week 4) was 48.8% in the oral-iron group (Figure 2). At the week 8, 12, 24, and 52 study visits, respectively, 11.1, 19.9, 25.9, and 28.7% of patients who had not responded by week 4 showed a response (Figure 3). On Kaplan-Meier analysis, there was a significant difference across all three treatment groups in terms of time to first Hb response after week 4 in the week 4 nonresponders (Figure 1b). The median time to first Hb response in the week 4 nonresponders was 92 days (95% CI 85, 168), 187 (168, 253), and 253 (169, 309) days in the high-ferritin FCM, low-ferritin FCM, and oral-iron groups, respectively. 

Overall, the cumulative rate of response (i.e., patients showing a response on at least one occasion) by week 52 was 83.0% (127/153) in the high-FCM group, 61.5% (91/148) in the low-FCM group, and 61.7% (185/300) in the oral-iron group (Supplemental Table 2). 

**Figure 4.**
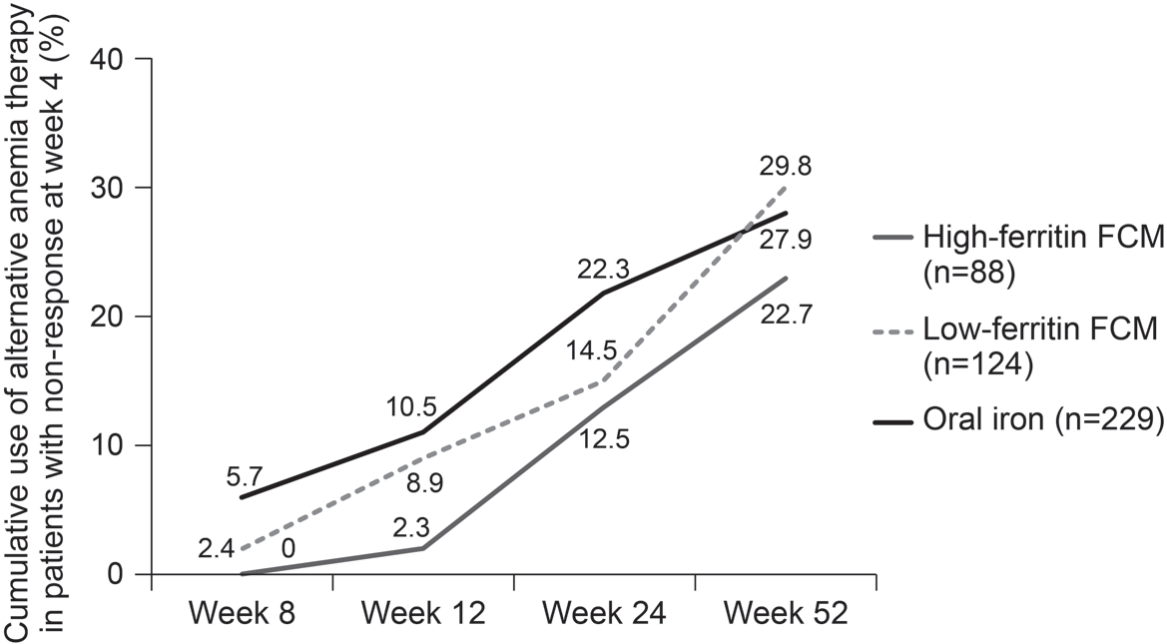
Cumulative use of alternative anemia therapy by weeks 8, 12, 24, and 52 in patients who were nonresponders at week 4.

Figure 4 shows the cumulative use of alternative anemia therapy, such as ESA or an alternative iron therapy, in patients who did not show an erythropoietic response by week 4 (alternative therapy was permitted only as rescue therapy up until week 8). By the end of the 52-week study, 27.9% (64/229) of patients who did not show an early response to oral iron had received alternative therapy. 

When baseline characteristics were compared between patients who did or did not respond to oral iron by week 4, there were no marked differences in age, gender, renal function (as assessed by eGFR), presence of diabetes or hypertension, or CRP level (Table 1). Use of concomitant medication that could potentially affect iron uptake from the gastrointestinal tract was also similar between early responders and nonresponders (Table 1). Mean baseline levels of Hb, ferritin, and TSAT were all lower in the responder cohort vs. nonresponders, with ferritin showing the most pronounced difference (mean 37 μg/L vs. 61 μg/L). Adherence to the oral iron dosing schedule throughout the study, as described in “Materials and methods”, was similar among patients who did or did not respond by week 4: mean (SD) adherence was 88.8 (16.3)% vs. 88.4 (18.5)%. There was a slightly higher adherence rate among the patients who responded to oral iron at some point during the study (mean (SD) 90.7 (14.0)%) compared to those who never responded (84.2 (23.5)%). 

## Discussion 

Oral iron supplementation is widely used in anemic ND-CKD patients with iron deficiency; however, results from this large, prospective study highlight the limited efficacy of this approach, with only 21.6% of patients having responded four weeks after continuous treatment with oral iron. Subsequently, there was a gradual increase in the proportion of responders to oral iron but this lessened over time, and more than half of the patients who did not respond by week 4 never achieved an Hb increase of at least 1 g/dL during the year-long study. 

The optimal timing to switch from oral iron to an alternative therapy in patients with ND-CKD, who do not show an increase in Hb, is not clear. In the current analysis, we elected to analyze responses by week 4 since after 1 month it would seem reasonable to monitor nonresponders more closely and to start considering an alternative intervention to avoid prolonged anemia. In this cohort, only a fifth had been switched to an alternative therapy by week 24, and fewer than 30% ever received alternative anemia therapy. It is possible that investigators may have kept patients on their randomized oral iron therapy in this controlled trial for longer than in routine practice, although a switch was permitted in the study protocol after week 8. The FIND-CKD study was not designed to test the efficacy of IV iron in patients not responding to oral iron therapy. Nevertheless, the strikingly higher rates of response in the high-ferritin FCM group highlight the potential benefit of switching oral iron nonresponders to IV iron therapy. Consistent with this, and beyond the area of CKD, a pooled analysis of data from the oral iron control arm of five randomized studies in patients with anemia of various etiologies (e.g., postpartum, heavy uterine bleeding, gastrointestinal disorders), reported in abstract form only, found that among patients without an Hb increase ≥ 1 g/dL after 2 weeks of oral iron therapy, 38.8% achieved a response after a switch to IV iron compared to only 10.2% who continued oral iron [18]. 

The observation that baseline Hb was lower in early responders is as expected. Experience with IV iron, where there is no barrier to uptake, has shown that the largest erythropoietic response occurs in patients with the most severe anemia [19, 20]. Equally, it is intuitive that patients with the worst iron status, based on ferritin and TSAT levels, will benefit most from iron supplementation. A previous analysis of erythropoietic response to oral iron therapy in patients on hemodialysis also demonstrated that baseline ferritin is significantly and inversely associated with likelihood of response [21]. Female gender is a widely-recognized risk factor for poor iron status, but its effect here may be muted by the generally elderly population, in whom menstrual blood loss would not apply. Baseline CRP level showed no association with early response to oral iron, so the effect of CRP on iron uptake was not sufficiently potent to influence the rate of erythropoiesis. Additionally, patients in this study generally had low CRP levels, as mandated by the study inclusion criteria. An effect of CRP may be more pronounced in the real-life setting where many patients have higher CRP levels. Another potential factor is the influence of concomitant medication. Antacids, H_2_ blockers, proton pump inhibitors, phosphate binders, and tetracycline antibiotics can all suppress uptake of oral iron from the gut [22]. Here, there was no substantial difference in the use of such agents between patients who did or did not respond early to oral iron. 

Low Hb and poor iron status are clinically-convenient indicators for the likelihood that patients may respond to oral iron therapy in this setting. Other possible predictors include hepcidin, a key regulator of iron metabolism, but hepcidin assays are not yet widely used, and data were not available in enough patients in the FIND-CKD study to be included in the analysis. The FIND-CKD study did not record other markers of red cell production, such as absolute or percentage reticulocyte count at baseline, which could be relevant [21, 22], but since the majority of nephrology centers do not measure reticulocyte parameters, these are unlikely to be relevant in routine clinical practice. 

The additional pill burden (usually three tablets a day) of oral iron therapy and frequent gastrointestinal side-effects both adversely affect adherence. In the context of this clinical trial, three-quarters of patients randomized to oral iron therapy showed good adherence, but in routine practice, rates may be lower. Level of adherence in the current population did not appear to account for differences between early responders and nonresponders, although there was a possible small effect of nonadherence in the longer term. 

A strength of the current study is that it included a comparatively large group of 300 ND-CKD patients treated with a typical oral iron regimen, with a high adherence rate. The limitations include the lack of a placebo-treated parallel control arm, which means that the findings do not take account of treatment-unrelated fluctuations in Hb levels, either upwards or downwards. The extent of within-patient variations in Hb have been documented, for example in patients on hemodialysis [23, 24], but applying estimates on the expected variations at 4-week intervals derived from other patient populations (e.g., hemodialysis patients, athletes, or healthy individuals) was not considered reliable. 

In conclusion, these results suggest a low rate of early response to oral iron therapy (~ 22%), with only approximately half of early nonresponders showing a subsequent response (49%). It should be noted that patients were not receiving ESA therapy, and these findings do not necessarily apply to ESA-treated individuals or to other settings. On the other hand, oral iron intolerance was an exclusion criteria so adherence to oral iron therapy is likely to have been greater in this controlled trial than in the real-life clinical setting. Thus, nonresponse to oral iron in routine practice due to poor adherence may have been underestimated. The data from this study also suggest that lower baseline levels of Hb, ferritin, and TSAT may predict a higher probability of an early erythropoietic response to oral iron, but further research is required to develop more sophisticated predictive models. 

## Funding 

This work was supported by Vifor Pharma, Glattbrugg, Switzerland. 

## Conflict of interest 

Iain C. Macdougall has received speakers’ fees, honoraria, and consultancy fees from several manufacturers of ESAs and IV iron, including Affymax, AMAG, Amgen, Ortho Biotech, Pharmacosmos, Hoffmann-La Roche, Takeda, and Vifor Pharma. Andreas H. Bock has received speakers honoraria and consultancy fees from Amgen, Hoffmann-La Roche, and Vifor Pharma. Fernando Carrera has no conflicts of interest to declare. Kai-Uwe Eckardt has received speaker’ fees and consultancy fees from several manufacturers of ESAs and IV iron, including Affymax, Amgen, Bayer, Johnson & Johnson, Hoffmann-La Roche, and Vifor Pharma. Carlo Gaillard has received speakers’ fees, honoraria, and consultancy fees from several manufacturers of ESAs and IV iron, including Amgen, Pharmacosmos, Hoffmann-La Roche, Takeda, and Vifor Pharma. David Van Wyck is an employee and stockholder of DaVita, Inc. Yvonne Meier, Amandine Perrin, and Sylvain Larroque are employees of Vifor Pharma. Simon D. Roger has received speakers’ fees, honoraria, and consultancy fees from several manufacturers of ESAs and IV iron, including Amgen, Hoffmann-La Roche, Janssen-Cilag, Novartis, Sandoz, Takeda, and Vifor Pharma. 

## Appendix

**The FIND-CKD Investigators**

**Australia**: Simon D Roger (Gosford), Alastair Gilles (Newcastle), Randall Faull (Adelaide), Nigel D Toussaint (Parkville), Lawrence McMahon (Box Hill), Michael Suranyi (Liverpool), David Mudge (Brisbane), Brian Hutchison (Perth), Ashley Irish (Perth), Peter Kerr (Clayton), Hemant Kulkarni (Perth and Armadale), Grahame Elder (Westmead), Margaret Jardine (Concord); **Austria**: Karl Lhotta (Feldkirch), Gert Mayer (Innsbruck); **Belgium**: Raymond Vanholder (Gent), Bart Dirk Maes (Roeselare), Pieter Evenepoel (Leuven), Frédéric Debelle (Baudour), Michel Jadoul (Brussels), Max Dratwa (Brussels); **Czech Republic**: Igor Macel (Zdar nad Sazavou), Milan Dunaj (Litomysl), Milan Kvapil (Praha), Petr Bucek (Frydek-Mistek), Jitka Rehorova (Brno), Ales Hruby (Slavkov u Brna), Václava Honová (Pizen), Lada Malanova (Pizen), Martin Lucak (Prague), Dalibor Lecian (Praha), Martin Jirovec (Marianske Lazne), Jiri Vlasak (Sokolov), Ivan Rychlik (Sokolov), Stanislav Surel (Brno); **Denmark**: Anne-Lise Kamper (Kobehavn), Ove Ostergaard (Roskilde), Gudrun K Steffensen (Frederica); **France**: Leila Chenine (Montpellier), Gabrial Choukroun (Amiens), Philippe Zaoui (Grenoble); **Germany**: Christoph Wanner (Würzburg), Wolfgang Backs (Hamburg), Uwe Kraatz (Demmin), Frank Dellanna (Düsseldorf), Klaus Busch (Dortmund), Tobias Marsen (Köln), Wolfgang Seeger (Berlin), Rainer Woitas (Bonn), Nicholas Obermueller (Frankfurt/Main), Thomas Haak (Bad Mergentheim), Stephan Lueders (Cloppenburg), Frank Pistrosch (Hoyerswerda), Eckhard Mueller (Benkastel-Kues), Peter R Mertens (Magdeburg), Werner Sutermer (Würzburg), Scott-Oliver Grebe (Wuppertal), Syrus Hafezi-Rachti (Mannheim-Käfertal), Silke Roeser (Eberswalde); **Greece**: Dimitrios Tsakiris (Thessaloniki), Dimitrios Memmos (Thessanloniki), Demetrios Vlachakos (Chaidari, Athens), Vassilis Vargemezis (Dragana, Alexandroupolis), Ioannis Stefanidis (Mezourlo, Larissa), Christos Syrganis (Volos), Polichronis Alivanis (Rhodes), Ioannis Papadakis (Athens), Nickolaos Papagalanis (Athens), Aimilios Andrikos (Joannina), Dimitrios Goumenos (Rios Patras), Kostas Siamopoulos (Ioannina), Charikelia Gouva (Arta), Gabriel Papadakis (Peireus), Ioannis Boletis (Athens), Myrsini Tsimnadi-Spanoudaki (Vestos), Dimitrios Stamatiades (Serres), Kyriaki Stamatelou (Athens), Spyridon Moutafis (Athens); **Italy**: Francesco Locatelli (Lecco), Antonio Santoro (Bologna), Francesco Quarello (Torino), Giuseppe Remuzzi (Bergamo), Salvatore Coppola (Piedmonte Matese), Rosella Ferraro Mortellaro (Dan Daniele del Friuli), Andrea Icardi (Arenzano), Giacomo Colussi (Milan), Franco Della Grotta (Anzio), Luigi Lombardi (Catanzaro), Maurizio Gallieni (Milano), Giuseppe Villa (Pavia), Giuseppe Grandaliano (Foggia); **The Netherlands**: Carlo Gaillard (Amersfoort and Amsterdam), Sebastiaan Huisman (Delft), Jos Barendregt (Apeldoorn), Peter JH Smak Gregoor (Dordrecht); **Norway**: Cecilia Oien (Trondheim); **Poland**: Boleslaw Rutkowski (Gdansk), Robert Malecki (Warszawa), Michal Nowicki (Lodz), Przemyslaw Rutkowski (Starogard Gdanski), Kryzsztof Marczewski (Zamosc), Michal Mysliwiec (Bialystok), Antoni Sydor (Tarnow), Jacek Rysz (Lodz), Andrzej Rydzewski (Warszawa), Marian Klinger (Wroclaw), Rafal Wnuk (Dabrowa Gornicza), Piotr Kozminski (Mlawa), Anna Nocon (Wroclaw), Kazimierz Ciechanowski (Szczecin); **Portugal**: Pedro Correia (Amadora), Fernando Neves (Lisboa), José Barata (Carnaxide); **Romania**: Gabriel Mircescu (Bucuresti), Mihai Voiculescu (Bucuresti), Gheorghe Gluhovschi (Timisoara), Eugen Mota (Craiova); **Spain**: Angel Luís Martín De Francisco (Santander), Alberto Torre (Madrid), Alba Herreros (Barcelona), José Luño (Madrid), E Gruss (Alcorcón), Judith Martins (Getafe [Madrid]), Marti Vallés (Girona), Julio Pascual (Barcelona); **Sweden**: Peter Bárány (Stockholm); **Switzerland**: Patrice M Ambuehl (Zürich); **Turkey**: Sehsuvar Erturk (Ankara), Mustafa Arici (Ankara), Saime Paydas (Adnana), Zeki Soypacaci (Izmir), Taner Camsari (Izmir), Sedat Ustundag (Edirne); **United Kingdom**: Iain C Macdougall (London), Mark E Thomas (Birmingham), Richard J D’Souza (Exeter), Jo E Taylor (Dorchester), Nicholas R Pritchard (Cambridge), Robin Jeffery (Bradford), Stephen G Riley (Cardiff), Deepak Bhatnagar (Oldham), Sunil Bhandari (Hull), David Reaich (Middlesborough), Paul E Stevens (Canterbury), Mohsen El Kossi (Doncaster), Simon Roe (Nottingham), Brian Camilleri (Ipswich), Aimun Ahmed (Preston), Arif Khwaja (Sheffield), Barbara Thompson (Stevenage), Debasish Banerjee (London), Johann Nicholas (Wolverhampton), Alistair Hutchison (Manchester), Richard Borrows (Birmingham). 

**Table 1.** Demographic and baseline characteristics according to response or nonresponse to oral iron therapy by week 4 (ITT population).

	Responders (n = 63)	Nonresponders (n = 229)
Age (years), mean (SD)	72 (14)	68 (14)
Weight (kg), mean (SD)	77 (17)	79 (18)
BMI (kg/m^2^), mean (SD)	29.0 (5.5)	29.1 (6.2)
Female, n (%)	39 (62)	145 (63)
White, n (%)	60 (95.2)	216 (94.3)
Hb (g/dL), mean (SD)	9.9 (0.7)	10.5 (0.6)
Ferritin (μg/L), mean (SD)	37 (33)	61 (39)
TSAT (%), mean (SD)	12 (6)	17 (8)
eGFR (mL/min/1.73m^2^), mean (SD)	34.1 (11.1)	31.7 (11.4)
CRP (mg/L), mean (SD)	4.5 (4.3)	5.4 (6.6)
Diabetes, n (%)	39 (61.9)	148 (64.6)
Hypertension, n (%)	14 (22.2)	55 (24.0)
Concomitant medication, n (%)		
Proton pump inhibitors	33 (52.4)	107 (46.7)
H_2_-receptor antagonists	3 (4.8)	5 (2.2)
Antacids	5 (7.9)	31 (13.5)
Any drug for excess gastric acid^a^	36 (57.1)	12 (53.3)
Phosphate-binders	0	7 (3.1)

^a^Proton pump inhibitors, H_2_-receptor antagonists, or antacids. Response was defined as a maximum increase in Hb from baseline of ≥ 1 g/dL, excluding patients who started alternative anemia management. BMI = body mass index; CRP = C-reactive protein; eGFR = estimated glomerular filtration rate; Hb = hemoglobin; SD = standard deviation; TSAT = transferrin saturation.

**Supplemental Table 1.** Key demographic and baseline characteristics in patients with (a) centrally-read Hb values at baseline and at week 4, or (b) with centrally-read Hb at baseline and week 4, or locally-read Hb values if missing.

Parameter	Treatment group	Central values only		Central or local values	
		n	Mean (95% CI)	n	Mean (95% CI)
Age (years)	Oral iron	221	68.8 (67.1; 70.6)	292	69.0 (67.5; 70.6)
	High-dose FCM	118	69.6 (67.3; 71.8)	149	69.3 (67.3; 71.4)
	Low-dose FCM	120	67.8 (65.3; 70.4)	144	67.8 (65.6; 70.0)
	Total	459	68.8 (67.5; 70.0)	585	68.8 (67.7; 69.9)
Male %	Oral iron	221	36.2 (29.9; 42.9)	292	37.0 (31.4; 42.8)
	High-dose FCM	118	39.8 (30.9; 49.3)	149	40.9 (33.0; 49.3)
	Low-dose FCM	120	37.5 (28.8; 46.8)	144	36.8 (28.9; 45.2)
	Total	459	37.5 (33.0; 42.1)	585	37.9 (34.0; 42.0)
Weight (kg)	Oral iron	221	78.6 (76.1; 81.0)	292	78.3 (76.2; 80.4)
	High-dose FCM	118	80.1 (76.7; 83.6)	149	79.0 (76.1; 82.0)
	Low-dose FCM	120	81.8 (78.5; 85.1)	144	81.2 (78.3; 84.0)
	Total	459	79.8 (78.1; 81.5)	585	79.2 (77.7; 80.6)
Hb (g/dL)	Oral iron	221	10.4 (10.3; 10.5)	292	10.4 (10.3; 10.5)
	High-dose FCM	118	10.3 (10.2; 10.5)	149	10.3 (10.2; 10.4))
	Low-dose FCM	120	10.4 (10.3; 10.6)	144	10.4 (10.3; 10.6)
	Total	459	10.4 (10.3; 10.5)	585	10.4 (10.3; 10.4)
Ferritin (μg/L)	Oral iron	221	56.3 (51.0; 61.6)	276	56.1 (51.5; 60.8)
	High-dose FCM	118	57.1 (48.8; 65.5)	142	55.3 (48.0; 62.6)
	Low-dose FCM	120	52.5 (45.0; 60.1)	141	53.8 (46.1; 61.5)
	Total	459	55.5 (51.7; 59.4)	559	55.3 (51.8; 58.8)
TSAT (%)	Oral iron	209	15.8 (14.7; 16.8)	279	15.6 (14.7; 16.5)
	High-dose FCM	110	14.3 (12.9; 15.6)	140	16.0 (13.2; 18.8)
	Low-dose FCM	116	15.7 (14.2; 17.2)	140	16.1 (14.7; 17.5)
	Total	435	15.4 (14.6; 16.1)	559	15.8 (14.9; 16.7)

Hb = hemoglobin; SD = standard deviation; TSAT = transferrin saturation.

**Supplemental Table 2.** Cumulative response rates to week 52 according to treatment group, n (%) (ITT population).

	Oral iron (n = 300)	High-ferritin FCM (n = 153)	Low-ferritin FCM (n = 148)
By week 4	63/292 (21.6)	61/149 (40.9)	20/144 (13.9)
By week 8	89/299 (29.8)	89/153 (58.2)	41/147 (27.9)
By week 12	111/300 (37.0)	97/153 (63.4)	54/148 (36.5)
By week 24	153/300 (51.0)	120/153 (78.4)	79/148 (53.4)
By week 52	185/300 (61.7)	127/153 (83.0)	91/148 (61.5)

Response was defined as a maximum increase in Hb from baseline of ≥1 g/dL, excluding patients who started an alternative anemia management or discontinued study drug.
